# School Siting Near Industrial Chemical Facilities: Findings from the U.S. Chemical Safety Board’s Investigation of the West Fertilizer Explosion

**DOI:** 10.1289/EHP132

**Published:** 2016-08-02

**Authors:** Veronica A. Tinney, Jerad M. Denton, Lucy Sciallo-Tyler, Jerome A. Paulson

**Affiliations:** 1 U.S. Chemical Safety and Hazard Investigation Board, Washington, DC, USA; 2 Department of Pediatrics, George Washington University School of Medicine and Health Sciences, Washington, DC, USA; 3 Department of Environmental and Occupational Health, George Washington University Milken Institute School of Public Health, Washington, DC, USA

## Abstract

**Background:**

The U.S. Chemical Safety and Hazard Investigation Board (CSB) investigated the 17 April 2013 explosion at the West Fertilizer Company (WFC) that resulted in 15 fatalities, more than 260 injuries, and damage to more than 150 buildings. Among these structures were four nearby school buildings cumulatively housing children in grades kindergarten–12, a nursing care facility, and an apartment complex. The incident occurred during the evening when school was not in session, which reduced the number of injuries.

**Objectives:**

The goal of this commentary is to illustrate the consequences of siting schools near facilities that store or use hazardous chemicals, and highlight the need for additional regulations to prevent future siting of schools near these facilities.

**Discussion:**

We summarize the findings of the CSB’s investigation related to the damaged school buildings and the lack of regulation surrounding the siting of schools near facilities that store hazardous chemicals.

**Conclusions:**

In light of the current lack of federal authority for oversight of land use near educational institutions, state and local governments should take a proactive role in promulgating state regulations that prohibit the siting of public receptors, such as buildings occupied by children, near facilities that store hazardous chemicals.

**Citation:**

Tinney VA, Denton JM, Sciallo-Tyler L, Paulson JA. 2016. School siting near industrial chemical facilities: findings from the U.S. Chemical Safety Board’s investigation of the West Fertilizer Explosion. Environ Health Perspect 124:1493–1496; http://dx.doi.org/10.1289/EHP132

## Introduction

On 17 April 2013, an explosion that occurred at the West Fertilizer Company (WFC) in West, Texas, resulted in the death of 15 persons and hundreds of injuries. The U.S. Chemical Safety and Hazard Investigation Board (CSB), an independent U.S. federal agency charged with investigating industrial chemical accidents and issuing recommendations aimed at preventing and mitigating their recurrence, conducted a detailed review of the devastating explosion. The CSB’s final investigation report, released in January 2016, illustrates the severe public health impacts of chemical incidents when they occur at fixed facilities that neighbor residential communities (CSB 2016).

**Figure 1:**
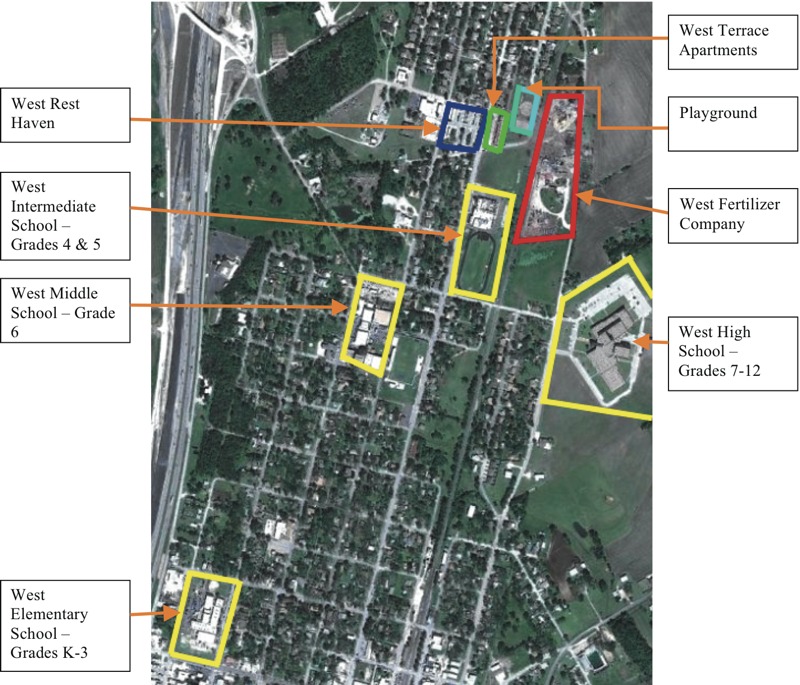
Map Showing Proximity of the WFC Facility to Schools and Other Public Structures (Source: Image © 2009 Google Earth, DigitalGlobe, with additional information provided by Greater Waco Chamber).

The explosion occurred at 1951 hours, only 20 minutes after the WFC fire was observed and reported to the fire department. The explosion of fertilizer-grade ammonium nitrate (FGAN)—with an explosive energy equivalent to cause the damage of 12.5 tons of TNT (2,4,6-trinitrotoluene)—fatally injured 12 emergency responders and three members of the community, and caused > 260 people to seek treatment for injuries. More than 150 offsite buildings were rendered uninhabitable following the incident. Among these structures were those of the nearby West Intermediate School and the West High School, located approximately 550 and 1,150 feet away, respectively, as shown in Figure 1. The siting of schools near facilities that store or produce hazardous chemicals is not unique to West, Texas. In the state of Texas alone, the CSB found that 19 (47.5%) of the 40 facilities storing FGAN are located within 0.5 miles of an elementary school, secondary school, or high school. One school identified was only 0.12 mile from a FGAN facility, which is closer than the schools damaged in West. The CSB has identified a lack of safe land use planning as a contributing factor to the severity of the consequences in 13 of its prior investigations.

Fortunately, the incident occurred in the evening, when school was not in session. All other conditions unchanged, had the fire and subsequent explosion occurred during the school day or when an evening activity or sporting event was taking place, it is likely that the injuries and fatalities would have been significantly greater, especially given the short time (20 minutes) that elapsed between the fire and the explosion. The total that could have been exposed at all four schools, assuming full attendance, was 1,486 students and 191 staff members, with 665 students and 86 staff combined at the Intermediate School and the High School, both of which sustained the most damage.

**Figure 2:**
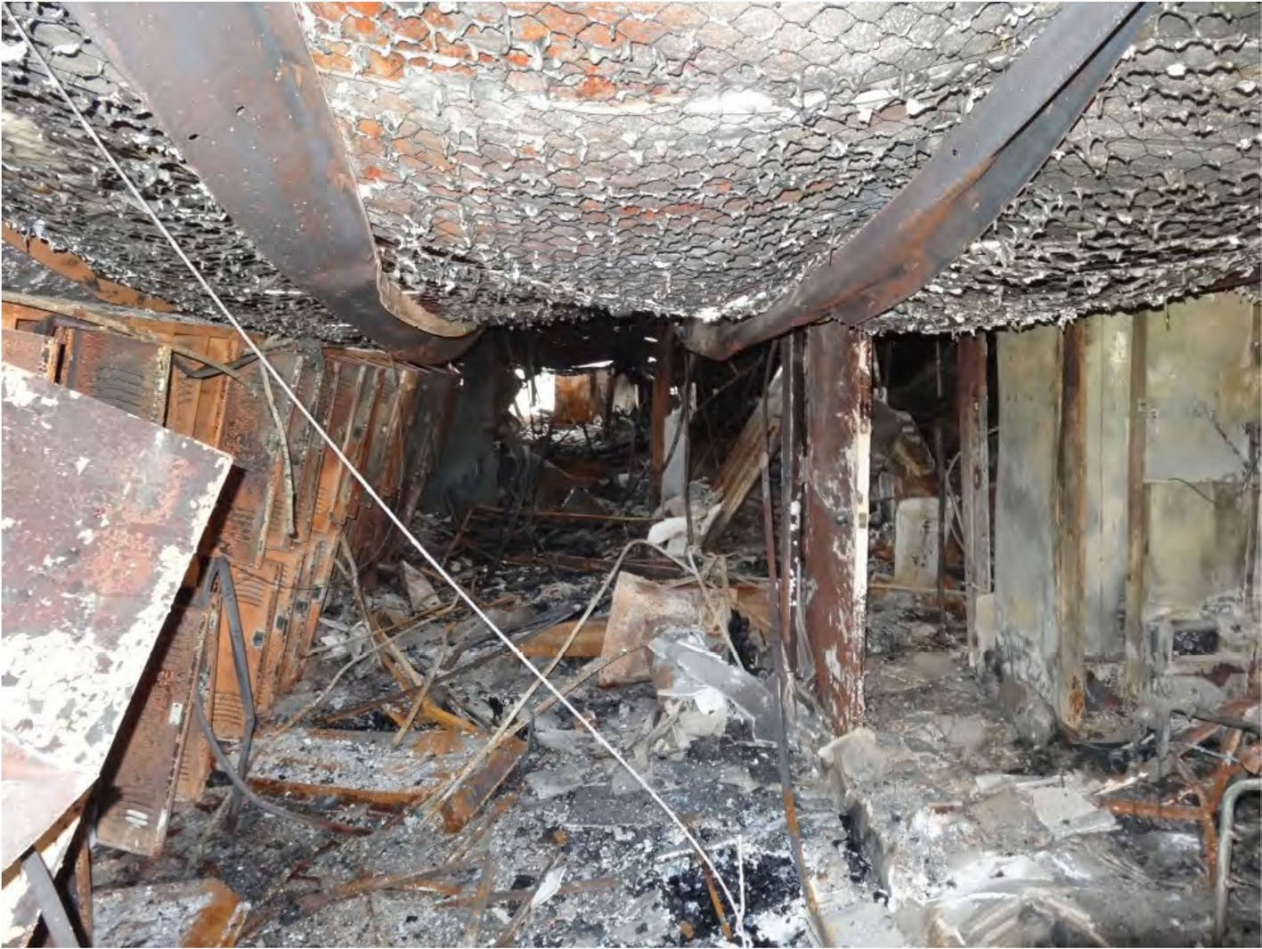
Interior of Burned Northeast Section of West Intermediate School (Source: ABS Consulting 2015).

Blast overpressure from the explosion, as well as fires that began postexplosion, caused damage to the West Elementary School, High School, Middle School, and Intermediate School. Damage surveys showed that debris accumulated in the hallways and ceilings in several classrooms and the gymnasium collapsed at the West Intermediate School, as shown in Figure 2. Following the blast wave, a fire also started at West Intermediate School, which would have exposed students and staff to heat and smoke. The ceiling, light fixtures, and other debris were thrown onto the desks of one classroom. Glazing hazards—or evidence of flying glass fragments—were found in the schools a significant distance from broken windows. The CSB commissioned blast modeling experts to examine structural damage and estimate overpressures at varying locations in all directions from the center of the explosion. External pressures measured between 0.4 and 1 lb/in^2^ (psi) [2.76 kPa (kilopascals)] for the West High School and 0.8 to 2.0 psi (5.52–13.79 kPa) for the Intermediate School. Injuries such as lacerations from glass and flying debris are commonly associated with these overpressures. The level of structural damage within the schools ranged from light, repairable damage and window glazing to large deformation of structural components. The more severe damage is usually associated with serious injuries to occupants, and the CSB estimated that 10-40% of occupants would have suffered fatal injuries (ABS Consulting 2015). Any students and staff present would have been covered in debris and would have had to climb over the debris to reach the exit. Due to the extent of the damage, the Intermediate and High Schools were demolished, with much of the Middle School demolished as well. The full details and consequences of the incident can be found in the CSB’s final investigation report (CSB 2016).

The tragedy at WFC caused many to wonder why a community was located so close to an FGAN storage facility. The WFC began operations in 1962; as the City of West developed over the years, it expanded toward the facility without any state or local zoning regulations to govern the separation between WFC and community structures. The schools closest to the WFC that sustained the most damage—the Intermediate and the High School—were built in 1985 and 2000, respectively, after the fertilizer storage facility began operation. Texas, like many states in the United States, has no state regulations relating to siting schools near hazardous facilities; as a result, the school system was not prohibited from siting its buildings near a facility that stored hazardous chemicals.

## Discussion

Several studies have attempted to quantify the risk of exposure to students from chemical incidents, as well as the frequency with which chemical incidents injure children at school. A study using the Hazardous Substances Emergency Events Surveillance (HSEES) system, coordinated by the Agency for Toxic Substances and Disease Registry (ATSDR) found that between 1999 and 2008, 11% (1,730) of the 15,506 persons injured from chemical incidents were students exposed at school (Duncan et al. 2015); however, this analysis and other earlier analyses do not distinguish students injured by incidents such as school laboratory accidents, intentional acts, and offsite consequences from those injured by fixed industrial facilities (Wattigney et al. 2008). Data from the combined HSEES annual reports between 2003 and 2009 noted that of the events for which the ATSDR was able to geocode, approximately 5,962 of the reported 53,036 events occurred within 0.25 mile of a school (ATSDR 2004, 2005, 2006, 2007, 2009, 2010).

Analysis using information submitted to the U.S. Environmental Protection Agency’s (EPA) Risk Management Program (RMP) characterizes the risk imposed to students from sources near educational institutions. The RMP requires facilities with more than a specified quantity of a specified substance to report information to the U.S. EPA and implement a risk management program. Part of this program includes a type of hazard analysis called a vulnerability zone, which identifies the geographic area and population that would be affected should a hazardous substance release occur (U.S. EPA 1987). Using RMP information collected by EPA, the Center for Effective Government has estimated that 19.6 million (36.6%) of 53.6 million children attend schools located in the vulnerability zone of fixed facilities that report to the RMP (Frank and Moulton 2014).

Proximity to industrial facilities also includes potential exposures to hazardous chemicals accidental air releases. For example, the CSB’s investigation of the 2012 fire at the Chevron Refinery in Richmond, California, found that exposure to the particulates resulting from the plume caused approximately 15,000 people to seek medical attention (CSB 2015). Legot et al. (2010) looked at facilities with the highest releases of air toxics, gathered from the U.S. EPA Toxics Release Inventory data, for five chemicals that are known developmental toxins (lead, mercury, carbon disulfide, manganese, and toluene) and found that 1,977 schools were located within approximately a 2-mile radius of 305 facilities, putting approximately 964,525 children at risk.

Currently no federal agency has the authority to prohibit school siting near hazardous facilities, or to consider potential environmental hazards of the site or adjacent site when siting schools. All states have compulsory education laws, and the overwhelming majority of students attend school outside the home. States typically delegate authority for decision making to the local education agency and for land use planning to local municipalities (NASBE 2016). In 2006, Rhode Island Legal Services (RILS) surveyed state laws, regulations, and policies to determine which states had codes or regulations in place to manage the siting of schools near hazardous facilities and other pollution sources. RILS found that at the time of the survey, 20 states had no policies that addressed the siting of schools near environmental hazards, including the assessment of potential school sites and their proximity to environmental hazards (RILS 2006). Further, only 14 state policies prohibit the siting of schools near hazards or pollution sources; the more common policy is only to require the consideration of siting factors. For example, California has established standards for selecting the location of new schools, including prohibiting the siting of schools near railroads, areas with heavy traffic, aboveground water or fuel storage tanks, aboveground or underground pipelines that pose a safety hazard, or hazardous waste disposal (California Department of Education 2015). In addition, California schools receiving state funding must perform an environmental assessment that considers the threat of a nearby release of hazardous material (California Education Code 1996). Though most of these policies are in place under state education codes and departments of education, environmental planners, educators, and public health professionals all have a role in influencing school siting policies and preventing school siting near hazardous facilities (Cohen 2010).

Though there is no federal agency with the authority to regulate school siting, the U.S. EPA was authorized by Congress to create voluntary school siting guidelines. The resulting School Siting Guidelines (U.S. EPA 2011) are the most robust that exist for considering environmental exposures in the siting of schools. Although these siting guidelines are indeed comprehensive, they are nonetheless voluntary. The guidelines are not intended to apply to existing schools, and only include the consideration of environmental and siting factors for new uses or new schools.

Though the U.S. EPA’s guidelines focus on exposure to environmental hazards and health risks, such as exposure to air pollution, they also cover physical hazards, such as fire or explosion. As they relate to large industrial facilities, the guidelines state that the screening perimeter for identifying large industrial facilities of interest is 0.5 mile. The World Health Organization (WHO), in their information series on school health, recommends a distance of 2 miles between schools and hazardous facilities (WHO 2003), which is consistent with the CSB’s observations of significant community damage in the City of West up to 2 miles away from the explosion epicenter. The potential safety hazards posed by these industrial facilities include explosions or fire. With regard to ameliorating these hazards, the guidelines recommend emergency shelter design incorporated into the new schools and the use of all-hazards emergency response plans. In terms of identifying and evaluating all large industrial facilities within a 0.5-mile radius, the guidelines state that the evaluation should include consulting air quality agencies.

Based on the findings of the West investigation and the identification of similar situations in Texas and the United States by others, we suggest that additional guidance include considering the physical properties, such as explosive or flammability characteristics, of the materials stored at these identified industrial facilities. The use of setback distances when considering the location of new schools would also help reduce exposure to physical hazards such as fire and explosion.

Actions to ensure awareness of chemical hazards near school buildings and communities before initiating new development can be taken by government agencies, community members, local emergency response officials and school officials. Schools should consult with local emergency response officials, such as local emergency planning committees or state emergency response officials, to better understand the characteristics of the chemicals present at industrial facilities in their locale and to be included in emergency response plans and practice activities. Operators and owners of facilities should likewise engage in communicating the hazards to neighboring communities, and the U.S. EPA has developed, in its RMP program guidance, guidelines for facilities in providing data and information to the public (U.S. EPA 2004). Public information tools, such as geographic information systems, are available through the the U.S. EPA’s Risk-Screen Environmental Indicators (RSEI) program (https://www.epa.gov/rsei) and Toxics Release Inventory program (https://www.epa.gov/toxics-release-inventory-tri-program), which provide information and data on the location of facilities with hazardous chemicals required to report to the U.S. EPA under the Emergency Planning and Community Right-to-Know Act (EPCRA) (2011), as well as information on the risks associated with the chemicals in use at each facility. Armed with greater awareness and knowledge of hazards, local governments and land use planners can then make informed decisions and use a variety of regulatory tools to mitigate the potential offsite impacts of hazards at industrial facilities. This includes the use of protective zoning, which can restrict development in hazardous areas (Schwab 2010). Regulatory tools that have been used to manage land use hazards near transmission pipelines, which may be translated for use near chemical facilities, include low-density zoning requirements near facilities; the use of fire resistance in the building codes for public buildings; deed restrictions on development; and the use of setback distances near chemical facilities (Osland 2013).

## Conclusion

The CSB’s investigation of the WFC explosion highlights the devastation that can occur when schools and communities are located near facilities storing hazardous chemicals. In light of the current lack of federal authority for oversight of land use near educational institutions, states should take a proactive role in promulgating state regulations that prohibit the siting of schools near facilities that store hazardous chemicals. The CSB is in the process of undertaking researching issues concerning land use planning near industrial facilities. Such research will focus on documenting the extent of the problem and assessing the adequacy of existing regulations and policies related to land-use planning near chemical facilities. The safety of our communities is a shared responsibility and the CSB hopes that other federal agencies, and state and local authorities with FGAN in their jurisdictions, will learn from the lessons of the WFC investigation and partner with the CSB on outreach and advocacy activities to ensure that the places where our children learn are not vulnerable to the consequences of chemical accidents.
